# Demographic and Clinical Predictors of Disease Severity in Patients With Multiple Sclerosis: A Retrospective Cross-Sectional Analysis

**DOI:** 10.7759/cureus.46873

**Published:** 2023-10-11

**Authors:** Awad M Almuklass, Ghidaa T Gosty, Eman B Alotaibi, Bushra T Alharbi, Reem A AlShayea, Ahmed B Aba Alkhail, Mashal B Abaalkhail

**Affiliations:** 1 Department of Basic Medical Sciences, King Saud Bin Abdulaziz University for Health Sciences College of Medicine, Riyadh, SAU; 2 Department of Research, King Abdullah International Medical Research Center, Riyadh, SAU; 3 Department of Neurology, King Saud Bin Abdulaziz University for Health Sciences College of Medicine, Riyadh, SAU; 4 College of Medicine, King Saud University, Riyadh, SAU

**Keywords:** hospital visits, methylprednisolone, body mass index, hospitalization, multiple sclerosis

## Abstract

Objectives: Multiple sclerosis (MS) is a disease of the central nervous system (CNS). Several factors, including sex, body mass index (BMI), disease duration, and age of onset, have been identified as predictors of disease severity. This study investigated the association between the aforementioned factors and MS severity, measured by the number of hospital visits and admissions, length of stay, and frequency of methylprednisolone use.

Methods: This retrospective cross-sectional analysis used data obtained from BESTCare at the King Abdulaziz Medical City (KAMC). A total of 272 patients with MS and their demographic and clinical characteristics were included.

Results: The study population consisted of 68.75% (n = 187) females and 31.25% (n = 85) males. The regression analyses indicated that disease duration was a significant predictor of the number of hospital visits and admissions (p < 0.01). The study found a significant association between BMI (unstandardized beta (B) = -0.25, 95% confidence interval (CI) = -0.47, -0.02, p = 0.033), age at diagnosis (unstandardized beta (B) = 0.15, 95% CI = 0.001, 0.31, p = 0.048), and length of hospital stay. Additionally, there was a significant correlation between disease duration and the number of methylprednisolone doses (unstandardized beta (B) = 0.45, 95% CI = 0.01, 0.89, p = 0.045).

Conclusion: Disease duration was found to be a significant predictor of hospital visits, admissions, and methylprednisolone use, while sex and BMI did not contribute to the variation in these outcomes. However, BMI and age of onset were significantly associated with length of hospital stay.

## Introduction

Multiple sclerosis (MS) is a chronic inflammatory disease of the central nervous system (CNS) that affects approximately 2.8 million people worldwide [1]. While the etiology and pathogenesis of MS are not fully understood, genetic and environmental factors are thought to play a role [2,3]. Multiple sclerosis (MS) is a complex disease that presents with diverse clinical symptoms. The latest MS clinical course classification outlines two distinct syndromes: clinically isolated syndrome (CIS) and radiologically isolated syndrome (RIS) [4]. Clinically isolated syndrome denotes the initial presentation of inflammatory demyelination and does not meet the diagnostic criteria for MS. Radiologically isolated syndrome is defined by the presence of radiological markers of demyelination without clinical symptoms. The clinical course of MS can be categorized into relapsing-remitting and progressive disease types. The new classification system encompasses temporal and dynamic information related to disease processes [5].

Several factors, including sex, body mass index (BMI), disease duration, and age at onset, have been identified as potential predictors of disease severity. Early childhood and adolescent obesity are significant risk factors for susceptibility [6,7]. Several studies have found a significant association between obesity and an increased risk of multiple sclerosis (MS), with a higher risk seen in females [7-9]. Obesity is linked to accelerated progression from a clinically isolated syndrome to MS and an increased frequency of relapses [10], although studies on the effects of body mass index (BMI) on MS severity have yielded mixed results [11-14]. Age of onset and sex are other factors that may influence MS severity, with older age of onset being associated with an unfavorable prognosis [15-17]. MS is more prevalent in females than in males, with a female-to-male ratio of approximately 3:1 [18]. However, according to a cohort study, the overall prognosis was more favorable for females, which can be attributed to the higher proportion of males with a primary progressive course [19]. Despite the growing body of research on the association of sex, body mass index (BMI), duration of disease, and age of onset with the severity of MS, the relationship between these factors is complex and may vary among individuals and populations. Moreover, the specific mechanisms underlying these associations are not fully understood. Thus, further research is needed to better understand these relationships and to develop more effective interventions for managing MS symptoms and preventing disabilities.

In this study, we aimed to investigate the association of sex, BMI, disease duration, and age of onset with the severity of MS measured by the number of hospital visits, frequency of methylprednisolone use, and length of hospital stay in patients with MS. By examining these variables, we hoped to gain a better understanding of the predictors of MS severity.

## Materials and methods

Study design and setting

This analytical retrospective cross-sectional chart review included patients diagnosed with clinically definite multiple sclerosis between 2015 and 2020 at King Abdulaziz Medical City (KAMC) in Riyadh, Saudi Arabia.

This study was approved by the Institutional Review Board (IRB) (number SP21R/181/04) at King Abdullah International Medical Research Center (KAIMRC) in Riyadh, Saudi Arabia, to ensure ethical compliance. After IRB approval, the research team acquired the data from the BESTCare system at the neurology department. Data were collected from medical records of the BESTCare system and then recorded on an Excel sheet (Microsoft Corp., Redmond, WA). Then, data were organized and prepared for visualization. Each patient was given a code only the investigators knew.

Patients and methods

The inclusion criteria for this study were patients diagnosed with clinically definite multiple sclerosis and aged >14 years. The exclusion criteria were immunocompromised MS patients (immunocompromised MS patients are patients who have other autoimmune diseases besides MS or a disease that might compromise their immune system), MS patients with cancer, and pregnant MS patients. A total of 272 patients with MS were included. For each patient, body mass index (BMI) was recorded within six months pre- or post-diagnosis. The disease duration was calculated by subtracting the current age from the age at the time of diagnosis. In addition, the number of inpatient admissions, number of emergency or outpatient visits, and total length of hospital stay were documented (length of hospital stay after the diagnosis with MS). The frequency of administration of methylprednisolone was also recorded.

Statistical analysis

Data were extracted, coded, and analyzed using the Statistical Package for the Social Sciences (SPSS) version 25 on Mac (IBM SPSS Statistics, Armonk, NY). The independent variables were sex, age, age at diagnosis, BMI, and disease duration. The dependent variables were the number of hospital visits, number of admissions, total length of hospital stay, and frequency of methylprednisolone use. Statistical significance was set at p < 0.05. Linear regression analyses were carried out to investigate correlations between variables, and multicollinear variables were removed. Any participant with an unreported BMI or with a data entry error was excluded from the analyses.

## Results

A total of 272 participants were included in the study. The sample consisted of 187 (68.75%) females and 85 (31.25%) males, with an average age of 33.77 ± 9.58 and an average age at diagnosis of 30.78 ± 9.27. Demographic characteristics are summarized in Table 1.

**Table 1:** Sociodemographic data of the participants (N = 272) SD: standard deviation

Characteristics	Number	Percent (%) = (number/272) × 100	Mean ± SD
Sex			
Female	187	68.75	
Male	85	31.25	
Age (years)			33.77 ± 9.58
Age at diagnosis (years)			30.78 ± 9.27

Linear regression analyses were conducted to predict the effects of age at diagnosis, sex, body mass index, and disease duration on the number of hospital visits, admissions, length of hospital stay, and methylprednisolone doses.

The regression model was significant for the number of visits (p < 0.001), with 19.37% of the variance explained by sex, BMI, age at diagnosis, and disease duration. However, only disease duration significantly predicted the number of visits (p < 0.001). Table 2 summarizes the linear regression model used to predict the number of visits.

**Table 2:** Linear regression model predicting the number of visits Results: F(4,267) = 16.03, p < 0.001, R2 = 0.19 Unstandardized regression equation: number of visits = 20.91 - 1.40 × sex - 0.20 × BMI - 0.009 × age at diagnosis + 2.81 × duration of disease CI: confidence interval, BMI: body mass index

Variable	Unstandardized beta (B)	Standard error	95% CI	β	t-value	p-value
Intercept	20.91	7.14	6.86, 34.96	0.00	2.93	0.004
Gender	-1.40	2.49	-6.30, 3.49	-0.03	-0.56	0.574
BMI	-0.20	0.19	-0.57, 0.17	-0.06	-1.06	0.291
Age at diagnosis (years)	-0.009	0.13	-0.26, 0.24	-0.004	-0.07	0.943
Duration of disease (years)	2.81	0.36	2.09, 3.52	0.43	7.75	<0.001

Similarly, the regression model was significant for the number of admissions (p = 0.032), with 3.87% of the variance explained by sex, BMI, age at diagnosis, and disease duration. However, only disease duration significantly predicted the number of admissions (p = 0.006). Table 3 summarizes the linear regression model used for predicting the number of admissions.

**Table 3:** Linear regression model predicting the number of admissions Results: F(4,267) = 2.69, p = 0.032, R2 = 0.04 Unstandardized regression equation: number of admissions = 1.32 - 0.003 × gender + 0.02 × BMI - 0.010 × age at diagnosis + 0.08 × duration of disease CI: confidence interval, BMI: body mass index

Variable	Unstandardized beta (B)	Standard error	95% CI	β	t-value	p-value
Intercept	1.32	0.54	0.26, 2.39	0.00	2.44	0.015
Gender	-0.003	0.19	-0.37, 0.37	-0.0009	-0.02	0.988
BMI	0.02	0.01	-0.004, 0.05	0.10	1.68	0.094
Age at diagnosis (years)	-0.010	0.010	-0.03, 0.009	-0.06	-1.01	0.315
Duration of disease (years)	0.08	0.03	0.02, 0.13	0.17	2.76	0.006

For the length of hospital stay, the regression model was insignificant (p = 0.131), indicating that sex, BMI, age at diagnosis, and duration of disease did not explain a significant proportion of the variation in length of hospital stay; however, BMI (p = 0.033) and age at diagnosis (p = 0.048) were significantly associated with length of hospital stay. Table 4 summarizes the linear regression model used to predict the length of hospital stay.

**Table 4:** Linear regression model predicting the length of stay Results: F(4,267) = 1.79, p = 0.131, R2 = 0.03 Unstandardized regression equation: length of stay = 8.75 - 0.29 × gender - 0.25 × BMI + 0.15 × age at diagnosis + 0.07 × duration of disease CI: confidence interval, BMI: body mass index

Variable	Unstandardized beta (B)	Standard error	95% CI	β	t-value	p-value
Intercept	8.75	4.35	0.19, 17.31	0.00	2.01	0.045
Gender	-0.29	1.51	-3.28, 2.69	-0.01	-0.19	0.846
BMI	-0.25	0.12	-0.47, -0.02	-0.13	-2.14	0.033
Age at diagnosis (years)	0.15	0.08	0.001, 0.31	0.12	1.98	0.048
Duration of disease (years)	0.07	0.22	-0.37, 0.50	0.02	0.30	0.766

For the number of methylprednisolone doses, the regression model was not significant (p = 0.194), indicating that sex, BMI, age at diagnosis, and disease duration did not explain a significant proportion of the variation in the number of methylprednisolone doses. However, disease duration was significantly correlated with the number of methylprednisolone doses (p = 0.045). Table 5 summarizes the linear regression model used to predict the number of methylprednisolone doses required.

**Table 5:** Linear regression model predicting NO Methylprednisolone doses Results: F(4,267) = 1.53, p = 0.194, R2 = 0.02 Unstandardized regression equation: methylprednisolone NO doses = 5.12 + 1.56 × gender + 0.08 × BMI - 0.07 × age at diagnosis + 0.45 × duration of disease CI: confidence interval, BMI: body mass index, NO: number of

Variable	Unstandardized beta (B)	Standard error	95% CI	β	t-value	p-value
Intercept	5.12	4.38	-3.51, 13.75	0.00	1.17	0.243
Gender	1.56	1.53	-1.45, 4.57	0.06	1.02	0.308
BMI	0.08	0.12	-0.15, 0.30	0.04	0.65	0.518
Age at diagnosis (years)	-0.07	0.08	-0.23, 0.08	-0.06	-0.94	0.349
Duration of disease (years)	0.45	0.22	0.01, 0.89	0.12	2.02	0.045

In summary, the regression model significantly predicted the number of visits (p < 0.001) and admissions (p = 0.032). Disease duration was a significant predictor of both outcomes. A one-unit increase in disease duration increased the number of visits by 2.81 units (p < 0.001) and the number of admissions by 0.08 units (p = 0.006). Additionally, although the overall model was insignificant, it is worth noting that disease duration was significantly associated with the number of methylprednisolone doses. Moreover, BMI and age at diagnosis were significantly associated with length of hospital stay, although the overall model was insignificant.

## Discussion

Multiple sclerosis is a heterogeneous neurological disease with varying degrees of severity [20]. The severity of MS can be influenced by various factors including smoking, pain, nocturia, depression, medication effects, and lesion location [20,21]. It has been suggested that the severity of MS can be influenced by sex, BMI, disease duration, and age of onset. The purpose of this study was to investigate the association of sex, body mass index (BMI), duration of disease, and age of onset with the severity of MS, as measured by the number of hospital visits, frequency of methylprednisolone use, and length of hospital stay.

The present study determined disease duration to be a significant predictor of the number of hospital visits, number of admissions, and frequency of methylprednisolone use. Sex, BMI, and age of onset did not significantly contribute to the variation in these outcomes. However, BMI and age of onset were significantly associated with length of hospital stay.

Several studies have demonstrated that obesity is associated with a twofold increased risk of MS, with estimates derived from population-based samples from Norway, Italy, Sweden, and the United States [7-9]. While the association was significant in both females and males, the risk was higher in females. A Kaiser Permanente study demonstrated that controlling for genetic factors did not change the association between increased BMI and MS [22]. Furthermore, the role of childhood obesity in the risk of both pediatric- and adult-onset MS has been confirmed in several studies [6,23]. Moreover, obesity has been found to correlate with accelerated progression from clinically isolated syndrome (CIS) to multiple sclerosis and an increased frequency of relapses [10].

With regard to the effect of BMI on MS severity, the current study revealed a significant correlation between BMI and length of hospital stay; however, this finding is contradictory to previous research showing that baseline BMI did not predict short-term changes in clinician- or patient-recorded disability in adult MS patients [11,12]. Similarly, BMI was also not found to be associated with disease activity in children with MS [24]. However, a small study demonstrated a modest correlation between obesity and the Expanded Disability Status Scale (EDSS) [25]. Moreover, two larger studies found that obesity correlated with increased disability severity in females, but not in males [13,14]. The significant association between BMI and length of hospital stay may be explained by the fact that BMI is an indicator of overall health and likely impacts the patient’s recovery time and ability to cope with the hospital environment.

This study revealed a noteworthy correlation between disease duration and MS severity, as indicated by the number of hospital visits and admissions. This finding is consistent with a previous study demonstrating that prolonged disease duration is linked to less favorable recovery from MS attacks [26]. This is likely due to the fact that as the disease progresses, patients may require more medical attention and interventions.

Relapsing-remitting MS age of onset has been previously shown to be associated with prognosis, with older patients having a higher risk of unfavorable outcomes and younger patients exhibiting a more favorable outcome and a slower rate of disability progression [16,17]. However, the current study did not reveal any significant association between age at onset and the tested outcomes, except for the length of hospital stay.

Sex is a potential factor that affects the severity of MS. However, this study found no significant association between sex and severity of MS. This is in contrast to a previous cohort study that demonstrated that females had a more favorable overall prognosis; however, this finding could be explained by the higher proportion of males with a primary progressive course of MS [19].

Despite the significant findings of this study, some limitations must be addressed. It was conducted at a single center and had a relatively small sample size, which may limit the generalizability of the results to other populations with different demographics and clinical characteristics. Moreover, the retrospective nature of the study is a limitation. Therefore, large-scale prospective studies are required to validate these findings.

## Conclusions

In conclusion, this study highlights the complexity of the relationship between various factors and the severity of MS. While disease duration was found to be a significant predictor of hospital visits, admissions, and methylprednisolone use, sex and BMI were not found to significantly contribute to the variation in these outcomes. However, BMI and age of onset were significantly associated with length of hospital stay. Further research is needed to better understand the complex interplay between various factors and MS severity and to explore other potential predictors that may affect the outcomes of interest.
